# Diet Is Associated with Frailty in Lung Cancer: A Possible Role of Gut Microbiota

**DOI:** 10.3390/nu15194298

**Published:** 2023-10-09

**Authors:** Ziyuan Li, Lei Qian, Jianghui Chu, Yuan Liu, Gusonghan Maitiniyazi, Yue Chen, Xinxin Cheng, Jianyun He, Lan Cheng, Minmin Ou, Jun Wang, Shufang Xia

**Affiliations:** 1 Wuxi School of Medicine, Jiangnan University, Wuxi 214122, China; liziyuan@stu.jiangnan.edu.cn (Z.L.); liuyuan@stu.jiangnan.edu.cn (Y.L.); chenyue@stu.jiangnan.edu.cn (Y.C.); chengxinxindeyx@163.com (X.C.); hejianyun@stu.jiangnan.edu.cn (J.H.); cl2773034328@163.com (L.C.); 1281210118@stu.jiangnan.edu.cn (M.O.); 2 Department of Rehabilitation, Wuxi Ninth People’s Hospital Affiliated to Soochow University, Wuxi 214063, China; leqian2020@163.com; 3 Department of Cardiothoracic Surgery, Affiliated Hospital of Jiangnan University, Wuxi 214125, China; jhchu2020@163.com; 4 Department of Nursing, College of Xinjiang Uyghur Medicine, Hetian 848000, China; gulsumm@163.com

**Keywords:** lung cancer, frailty, dietary quality, gut microbiota, nutrients

## Abstract

This study investigated the associations between diet and frailty in lung cancer patients and the potential role of the gut microbiota involved. We assessed dietary intake and frailty status in 231 lung cancer patients by 3-day, 24-h dietary recalls and Fried frailty criteria, respectively, and collected 50 fecal samples for next-generation sequencing. A total of 75 (32.5%) patients were frail, which might be related to significantly lower intake of energy, protein, carbohydrate, dietary fiber, niacin, leucine, some minerals, and a poorer dietary quality as indicated by the Chinese Healthy Eating Index (*p* < 0.05). Among these, carbohydrate (OR = 0.98; 95% CI 0.96–0.99; *p* = 0.010), calcium (OR = 0.99; 95% CI 0.99–1.00; *p* = 0.025), and selenium (OR = 1.03; 95% CI 1.00–1.06; *p* = 0.022) were all significantly associated with frailty. A multivariate logistic regression analysis showed that the mean risk of frailty was 0.94 times lower (95% CI 0.90–0.99; *p* = 0.009) among participants with higher CHEI scores. Additionally, the frail patients demonstrated significantly lower gut microbiota β diversity (*p* = 0.001) and higher relative abundance of Actinobacteriota (*p* = 0.033). Frailty in lung cancer patients might be associated with insufficient nutrients intake and a poor dietary quality through gut microbiota regulation.

## 1. Introduction

Lung cancer has become the second-highest incidence of cancer in the world and remains the leading cause of cancer-related deaths worldwide, accounting for 18% of global cancer deaths [1]. In China, lung cancer has the highest incidence and mortality among all cancer types [2]. Due to the disease diagnosis and treatment, the physiological reserves can decrease, which might cause a series of physical symptoms, such as falling or frailty [3]. Frailty, characterized by a decline in functioning across multiple inter-related physiological systems, including sedentariness, fatigue, weight loss, and poor muscle strength, accompanied by an increased vulnerability to stressors, is common in lung cancer patients [4]. It was estimated that the prevalence of frailty in lung cancer patients was 28–61% [5], which increased the risk of adverse outcomes, severely affected the prognosis of the disease, reduced quality of life and survival, and led to a threefold increase in mortality compared with non-frail patients [6]. Recently, frailty has been demonstrated as an independent predictor of prognosis in lung cancer patients [7]. However, the reality is that there are no effective treatments for frailty [8], and the most effective intervention is not yet known. Given the high prevalence of frailty in lung cancer patients and its severe outcomes, identification of risk factors for frailty in lung cancer and the possible underlying mechanisms are of great importance.

Thus far, it is not clear what drives frailty, and little is known about the risk factors that contribute to the development of the syndrome. The accumulated evidence suggested that the factors contributing to frailty might include sociodemographic characteristics (age, gender, BMI, and income), comorbidities, psychological conditions (depressive symptoms and cognition), and behavioral factors (lifestyles, smoking and alcohol use, nutritional status, and lack of physical activity). Among them, diet has been proposed as a key element in the development of frailty. Low intake of certain micronutrients and protein is associated with a higher risk of developing frailty [9]. In recent years, the focus on diet and chronic disease has shifted from specific nutrients to overall dietary quality and patterns measured by the predefined dietary scores based on dietary guidelines. An unhealthy dietary pattern (e.g., the Western diet) is related to an increased risk of frailty [10], while a healthy dietary pattern (diet rich in vegetables and fruits or the Mediterranean diet) is linked to a decreased risk of frailty. Due to the lack of professional dietary guidance and the side effects of chemotherapy, most lung cancer patients demonstrate low dietary quality or unreasonable dietary structure after disease diagnosis and during treatment [11], which might be responsible for frailty and lead to severe outcomes [12]. The Chinese Healthy Eating Index (CHEI), with good validity and reliability, is a tool for evaluating the overall dietary quality of the Chinese population based on 3-day, 24-h dietary recalls in accordance with the Dietary Guidelines for Chinese (DGC-2016) [13]. There are no studies on the relationship between frailty and CHEI-based dietary quality in lung cancer patients. Although frailty etiology has been attributed to multiple pathophysiological pathways, age-related muscle loss is considered to be of great importance in this syndrome [14], in which genetic, epigenetic, and environmental factors might be involved [15]. The gut microbiota is a complex and delicate commensal ecosystem, and its composition is affected by diet. Dietary ingredients and food additives have a substantial impact on the gut microbiota and thus affect human health [16]. Nowadays, a number of studies have directly or indirectly proved that the gut microbiota can affect the host’s muscle mass and function by regulating systemic inflammation and immunity, substance and energy metabolism, and insulin sensitivity through the gut–muscle axis [17]. Germ-free mice showed significant reduced muscle mass and strength [18]. Supplementing the symbiotics for critically ill patients could shorten the length of ICU stay and reduce muscle protein catabolism [19]. A case-control study also indicated that soy–whey blended protein attenuated muscle function through the gut microbiota in a subset of patients with hematological malignancies, who failed to enhance muscle function after hematopoietic stem cell transplantation [20]. Leucine, the essential branched chain amino acid (BCAA) for muscle health, must be obtained from the daily diet and is associated with the risk of frailty [21]. Recently, a growing focus revealed the interaction between leucine and the gut microbiota and subsequent different factors of muscle health. In lung cancer patients, whether leucine is a key element in the association in diet–gut microbiota–frailty is not clear.

As mentioned above, there might be complex and close relationships among diet, the gut microbiota, and frailty in lung cancer patients. Although a large number of studies on the above issue have been conducted in the elderly, and some in particular have confirmed the correlation between dietary quality and frailty [22], no research has explored the role of the gut microbiota in the association between diet and frailty in lung cancer patients. Therefore, the aim of this study was to determine the association between diet and frailty in lung cancer patients and the possible role of the gut microbiota involved.

## 2. Materials and Methods

### 2.1. Participants and Study Design

This cross-sectional research (Chinese Clinical Trials Registry ChiCTR 2200064105) was carried out at the Affiliated Hospital of Jiangnan University from September 2022 to February 2023 and approved by Medical Ethics Committee of Jiangnan University (JNU20220901IRB11). A total of 231 patients diagnosed with lung cancer were recruited based on the following inclusion criteria: (1) patients diagnosed with lung cancer by pathological examinations; (2) age ≥ 18 years old; (3) normal cognitive function and reading ability; (4) voluntary participation in the research. Participants who met the following exclusion criteria were excluded: (1) prior physician-diagnosed mental illness before or after lung cancer diagnosis; (2) diagnosed with other cancers; (3) diagnosed with digestive system diseases such as enteritis and gastritis; (4) use of chemotherapy medications, antibiotics, probiotics, or Chinese traditional medicine in the past 21 days; (5) other reasons that the researchers believed were not suitable for the research, such as following a vegetarian diet or consuming any nutritional supplements while participating in the study and actively trying to lose or gain weight. All participants provided written informed consent.

### 2.2. Sample Size Calculation

Based on the published literature, the prevalence of frailty in lung cancer patients was 45% [5]. We used the following formula for calculation: *n* = *Z*^2^ _α/2_ (1 − *p*) *p*/*δ* ^2^, where *α* = 0.05, *Z_α_*_/2_ = 1.96, and *p* is the prevalence of frailty in lung cancer patients, where *p* = 0.45, and *δ* is the permissible error, where *δ* = 0.15, *p* = 0.0675. The estimated minimum sample size was 208.

### 2.3. Frailty Assessment

Frailty status was evaluated using the Fried frailty criteria with a frailty score ranging from 0 to 5. It defines five criteria: (1) non-expected weight loss; (2) self-reporting fatigue for more than three days within a week; (3) muscle weakness; (4) low gait; (5) low physical activity level. The criteria for each of the frailty definitions are shown in Table 1 [23,24]. Patients with a score ≥ 3 were allocated into frailty group, and patients with a score < 3 were in the non-frailty group [25].

### 2.4. Dietary Intake Assessment and the Calculation of CHEI Component Scores

To assess the dietary intake of lung cancer patients, 3-day, 24-h dietary recall interviews were conducted. All participants were asked to describe in detail the types and amounts of foods consumed during the past 24 h. The trained nutrition education research assistants used food models and atlases to help participants recall their dietary intake through face-to-face interviews. Dietary data were entered into the Nutrition Calculator v2.8.1.8 (Institute of Nutrition and Food Safety, Chinese Center for Disease Control and Prevention, Beijing, China) to obtain nutritional intakes of each patient. The average nutrient intakes from the 3-day, 24-h dietary recalls were used as the final data for analysis.

CHEI score was calculated based on energy density (as amounts of per 1000 calories intake). The CHEI consists of 17 components, 12 of which are adequacy components, including total grains, whole grains and mixed beans, tubers, total vegetables, dark vegetables, fruits, dairy, soybeans, fish and seafood, seeds and nuts, and poultry and eggs. The remaining five groups are limitation components, including red meat, cooking oils, sodium, added sugars, and alcohol. Each component is scaled from zero to five (zero to ten for fruits, cooking oils, and sodium). The total CHEI score is a sum of scores of all 17 components, ranging from 0 to 100.

### 2.5. Plasma Leucine Determination

After obtaining the patients’ consent, a total of 83 fasting blood samples (52 from frail patients and 31 from non-frail patients) were collected, centrifuged at 3500 rpm for 5 min, and stored at −80 °C until further analysis. Plasma leucine levels were measured by ELISA kits according to the manufacturer’s instructions.

### 2.6. Gut Microbiota Analysis

Fifty fecal samples (14 from the frailty group and 36 from the non-frailty group) were collected from the patients before the chemotherapy for 16S rRNA sequencing (Shanghai Majorbio Bio-Pharm Technology Co., Ltd., Shanghai, China). DNA was extracted, followed by concentration and purity determination using Nanodrop 2000 UV-Vis spectrophotometer (Thermo Scientific, Wilmington, DE, USA) as well as integrity confirmation by 1% agarose gels. The purified DNA was amplified by the variable V3–V4 regions of 16S rRNA using primers 338F (5′-ACTCCTACGGGAGGCAGCAG-3′) and 806R (5′-GGACTACHVGGGTWTCTAAT-3′) with ABI GeneAmp^®^ Type 9700 PCR thermocycler (ABI, Carlsbad, CA, USA). Following the Illumina MiSeq PE300 platform (Illumina, San Diego, CA, USA), 16S Metagenomic Sequencing Library Preparation Program, amplicon multiplexing, merging, and sequencing were performed. The raw 16S rRNA gene-sequencing reads were demultiplexed and quality-filtered by fastp version 0.20.0 and then merged by FLASH version 1.2.7.

Operational taxonomic units (OTUs) with a 97% similarity cutoff were clustered with UPARSE version 7.1., in which chimeric sequences were identified and subsequently removed. The taxonomy of each OTU representative sequence was performed using RDP Classifier version 2.2 against the 16S rRNA database (e.g., Sliva v138), using a confidence threshold of 0.7. Alpha diversity was used to assess the complexity of species diversity in each sample with QIIME to calculate the Chao index. Mothur was used to generate a rarefaction curve with the Sobs index (the observed richness) on the OTU level to detect a reasonable amount of sequencing data. The rarefaction curves that tended to be flat suggested sufficient sequencing data. Beta diversity was evaluated through a principal coordinates analysis (PCoA) of weighted UniFrac distances based on the observed OTUs. The statistical significance was evaluated with an Adonis. In order to reveal the differences at the phylum and genus levels (*p*-values were corrected using the Benjamini–Hochberg false-discovery (FDR) method) and in the alpha diversity of bacterial communities, the Wilcoxon rank-sum test was used. To determine the statistically significant biomarkers and the dominant microorganisms within each group, Linear discriminant analysis Effect Size (LEfSe) with default criteria (*p* < 0.05 by a non-parametric factorial Kruskal–Wallis rank-sum test and linear discriminant analysis (LDA) score > 4) was used. Partial least squares–discriminant analysis (PLS-DA) was performed to identify gut microbiota that best discriminated the frail and non-frail participants. The analysis was conducted on the Majorbio Cloud Platform (www.majorbio.com, access date 15 May 2023).

### 2.7. Statistical Analysis

Statistical analysis was performed with SPSS 27.0 (IBM SPSS Inc., Chicago, IL, USA). Continuous variables were expressed as either median (25th, 75th percentile) or mean ± SD, while categorical variables were expressed as frequencies (N) and percentages (%). The Kolmogorov–Smirnov test was used to determine the normal distribution. For normally distributed data or non-normally distributed data, independent samples *t*-test and the Mann–Whitney U test were used to assess the differences between groups, respectively. Categorical variables were analyzed using the chi-square test or Fisher’s exact test. Univariate logistic regression analysis was conducted to reveal the possible influencing factors of frailty, and then, the variables with *p* < 0.05 were included in the subsequent multivariate logistic regression analysis, in which age, sex, BMI, and cancer stage were included as covariates in the models.

## 3. Results

### 3.1. Participants’ Characteristics

Among the 231 participants, 75 (32.5%) patients were allocated to the frailty group, while the remaining 156 (67.5%) patients were allocated to the non-frailty group. The characteristics of the frail and non-frail participants are shown in Table 2. Statistical significance was found in age (*p* = 0.025), BMI (*p* < 0.001), and cancer stage (*p* = 0.023), while no significant difference was revealed in sex, marital status, education level, residence, family monthly income, smoking status, drinking status, presence of comorbidities, and surgery and pathology type (*p* > 0.05).

### 3.2. Nutrient Intakes and CHEI Component Scores of the Patients

The dietary nutrient intakes of the frail and non-frail lung cancer patients are shown in Table 3. Compared to the non-frail patients, the frail patients had remarkably lower intakes of energy, protein, carbohydrates, dietary fiber, vitamin D, vitamin B1, vitamin B2, vitamin B6, vitamin C, niacin, calcium, phosphorus, potassium, magnesium, iron, zinc, iodine, selenium, copper, manganese, and leucine (*p* < 0.05), in which energy, protein, carbohydrates, dietary fiber, niacin, calcium, phosphorus, potassium, magnesium, zinc, iodine, selenium, copper, and manganese might be related to the frailty status in lung cancer patients according to the univariate logistic analysis (Table S1, *p* < 0.05). Multivariate logistic analysis further revealed that carbohydrates (OR = 0.97; 95% CI 0.96–0.99; *p* = 0.003), calcium (OR = 0.99; 95% CI 0.99–1.00; *p* = 0.025), and selenium (OR = 1.03; 95% CI 1.00–1.06; *p* = 0.022) were independent influencing factors for frailty (Table 4).

Regarding the total CHEI score and its component scores, the frail patients had significantly lower total CHEI score, dairy score, sodium score, and cooking oils score than the non-frail patients (Table 5, *p* < 0.05). Total CHEI score (OR = 0.94; 95% CI 0.91–0.97; *p* < 0.001) and dairy score (OR = 0.84; 95% CI 0.75–0.94; *p* = 0.002) were associated with frailty, respectively (Table 6), but only the total CHEI score was identified as an independent influencing factor for frailty in lung cancer patients (OR = 0.94; 95% CI 0.90–0.99; *p* = 0.009).

### 3.3. Plasma Leucine Levels of the Patients

**Figure 1:**
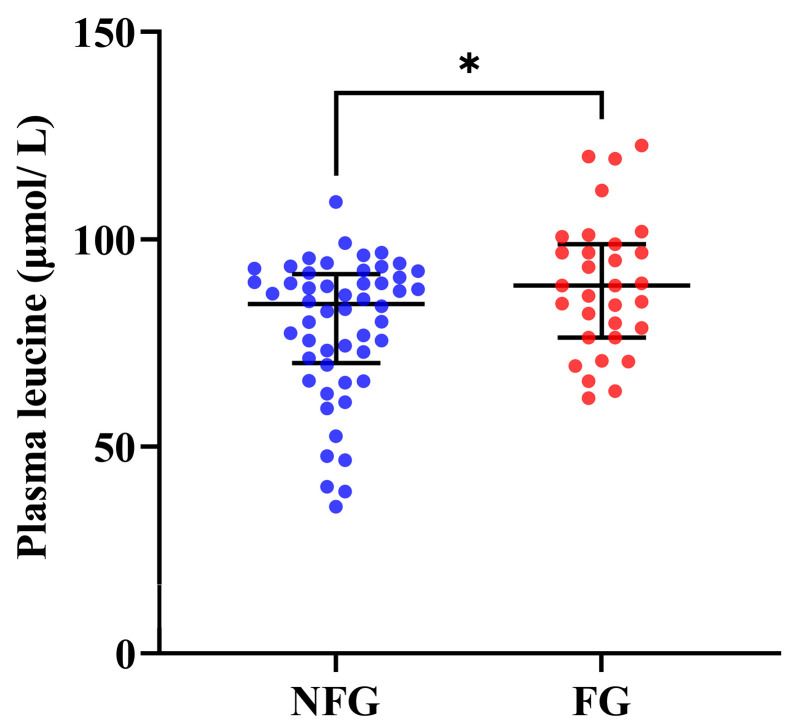
Plasma leucine levels between the frail and non-frail lung cancer patients. Data are shown as median (25th, 75th percentile). Statistical analysis was performed by the Mann–Whitney U test. * *p* < 0.05. FG, frailty group (*n* = 31); NFG, non-frailty group (*n* = 52).

As shown in Figure 1, the frail lung cancer patients demonstrated significantly lower plasma leucine levels than the non-frail patients (*p* = 0.036).

### 3.4. Gut Microbiota Diversity of the Patients

**Figure 2:**
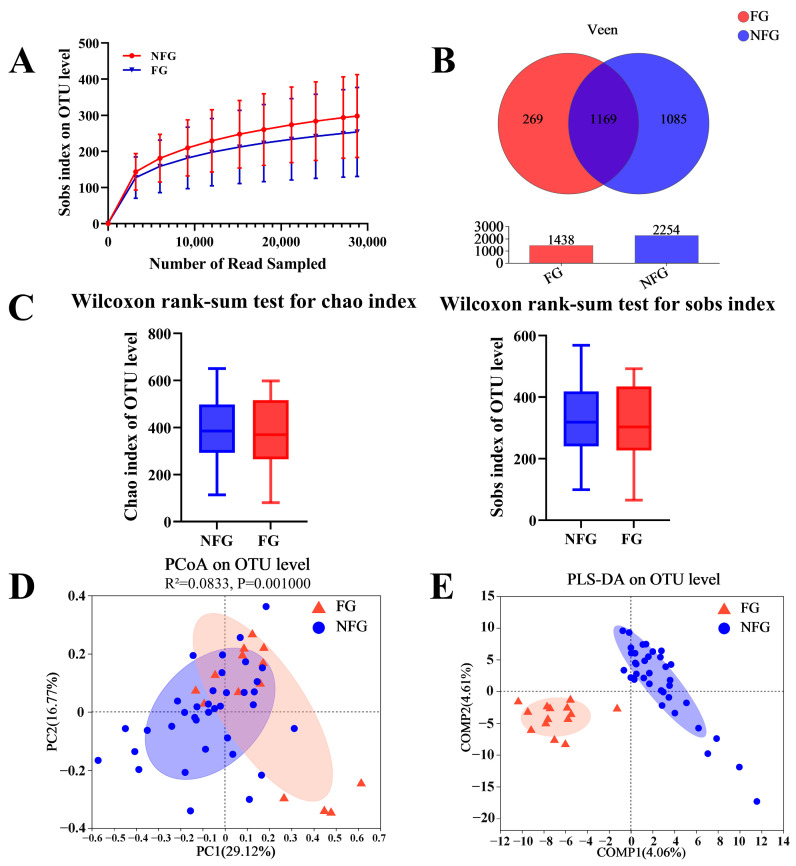
Differences in gut microbiota structure between the frail and non-frail lung cancer patients. (**A**) The rarefaction curves for the sequence depth assessment. (**B**) Venn diagram illustrating the shared number of operational taxonomic units by frail and non-frail patients. (**C**) Chao index and Sobs index for richness and evenness evaluation. (**D**) Weighted UniFrac distance-based principal coordinate analysis (PCoA). The percentages of variation explained by PC1 and PC2 are shown on the axis. Distances between the samples are based on similarity OTU composition (OTU similarity: 97%). The statistical significance was evaluated with analysis of similarities (Adonis). (**E**) Partial least squares–discriminant analysis (PLS-DA) of the two groups. Data are expressed as mean ± SD. FG, frailty group (*n* = 14); NFG, non-frailty group (*n* = 36).

Rarefaction curve analysis with 97% similarity of OTUs was used to confirm that the sequencing depth was sufficient for studying the microbial diversity (Figure 2A). The Veen diagram illustrated that 1169 OTUs were shared between the frail and non-frail patients (Figure 2B). Alpha diversity, suggesting the richness by Chao index and evenness by Sobs index, was not significantly different between the two groups (Figure 2C). However, the PCoA results demonstrated significant difference in the gut microbiota composition indicated by the weighted UniFrac distances (Adonis R^2^ = 0.0833, *p* = 0.001, Figure 2D). The PLS-DA plot also exhibited distinct separation between the two groups (Figure 2E). These results suggested significant differences in the gut microbiota composition between the frail and non-frail patients.

### 3.5. Gut Microbiota Composition of the Patients

**Figure 3:**
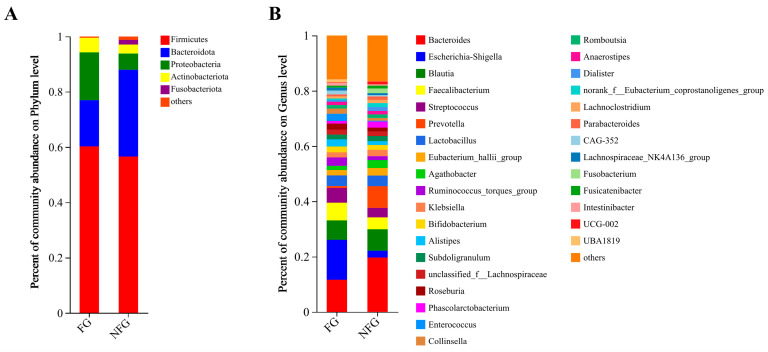
Gut microbiota composition in the frail and non-frail lung cancer patients. (**A**) The community structures of different microbes at the phylum level. (**B**) The community structures of different microbes at the genus level. FG, frailty group (*n* = 14); NFG, non-frailty group (*n* = 36).

**Figure 4:**
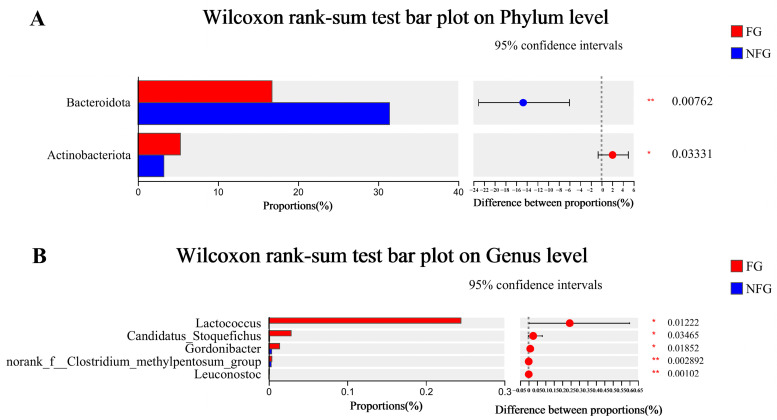
(**A**) Differentiated microbes at the phylum level. (**B**) Differentiated microbes at the genus level. FG, frailty group (*n* = 14); NFG, non-frailty group (*n* = 36). * *p* < 0.05, ** *p* < 0.01.

Firmicutes was the highest dominant phylum in both frail and non-frail patients, with an abundance of 60.22% and 56.54%, respectively. The second major phylum was Bacteroidota, with 16.73% and 31.42% abundance, respectively (Figure 3A). The dominant genera (>5%) were Bacteroides, Escherichia-Shigella, Blautia, Faecalibacterium, and Streptococcus in the two groups (Figure 3B). The frailty group exhibited a significantly lower relative abundance of Bacteroidota (*p* = 0.007) than the non-frailty group, while the relative abundance of Actinobacteriota was remarkably higher (*p* = 0.033, Figure 4A). The relative abundance of Lactococcus (*p* = 0.012), Candidatus_Stoquefichus (*p* = 0.034), Gordonibacter (*p* = 0.018), nonrank_f_Clostridum_methylpentosum_group (*p* = 0.003), and Leuconostoc (*p* = 0.001) were all significantly higher in the frail patients than those in the non-frail patients (Figure 4B).

**Figure 5:**
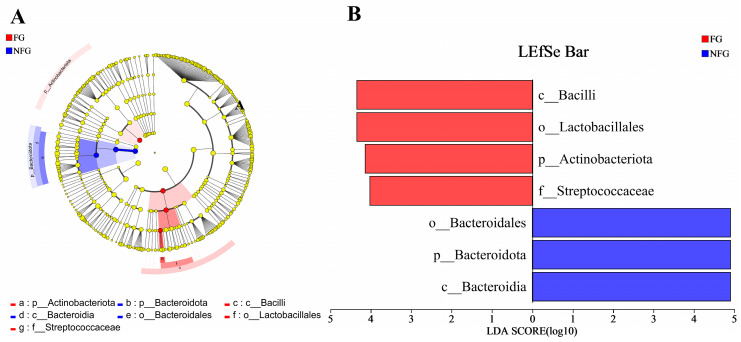
Differentiated microbes between the frail and non-frail lung cancer patients. (**A**) LEfSe analysis was used to distinguish the differential microbes between the FG and NFG patients. The different colored nodes represented microbial populations that were significantly enriched in the corresponding groups and showed significant differences between the groups. (**B**) LDA was performed, and only the microbiota with LDA scores of >4 are shown. FG, frailty group (*n* = 14); NFG, non-frailty group (*n* = 36).

LEfSe was used to reveal specific bacterial taxa that were significantly differentiated between the frail and non-frail groups (Figure 5). Seven differentiated taxa with a LDA score > 4 from phylum to genus were found, in which c_Bacilli (*p* = 0.018), o_Lactobacillales (*p* = 0.012), p_Actinobacteriota (*p* = 0.032), and f_Streptococcaceae (*p* = 0.018) were more abundant in the frail patients, while o_Bacteroidales (*p* = 0.007), p_Bacteroidota (*p* = 0.007), and c_Bacteroidia (*p* = 0.007) were more abundant in the non-frail patients.

## 4. Discussion

This cross-sectional study explored the associations between diet and frailty status in lung cancer patients and the potential role of gut microbiota involved. Our findings suggested that the frailty in lung cancer patients might be associated with insufficient intake of certain nutrients, especially carbohydrate, calcium, and selenium. Additionally, our results provided further evidence supporting the correlation between dietary quality and frailty. Specifically, a higher total CHEI score was negatively associated with frailty, indicating that a better dietary quality might be associated with a reduced risk of frailty. Moreover, the frail patients showed lower gut microbiota diversity and an unhealthy microbiota composition, which might be the potential reason for frailty development.

Frailty is a common clinical symptom among lung cancer patients, which can contribute to a series of adverse outcomes, including diminished quality of life and poor disease prognosis. In the present study, we found that the average age of the frail patients was significantly higher than that in the non-frail patients, which might be due to the fact that frailty has been considered to develop as a consequence of age-related decline in many physiological systems, which collectively results in vulnerability to sudden health status changes triggered by minor stressor events. Additionally, both cancer itself as well as the therapies offered could be significant additional stressors that challenge a patient’s physiologic reserve, leading to increased vulnerability to frailty [26]. The BMI of the frail patients was significantly lower than that of the non-frail patients. The previous study described a U-shaped association between BMI and physical frailty [27], suggesting both underweight and obesity, two unhealthy body weight statuses outside the normal range, were significantly associated with an increased risk of frailty [28].

In recent years, the relationship between diet and frailty has gained much attention. The observational and intervention studies showed promising results on the association between frailty and nutrients, including protein and some micronutrients, such as carotenoids and vitamins [29]. Protein is crucial for muscle mass and strength and might play a key role in frailty, as most of the studies indicated that high protein intake was inversely associated with frailty and individual frailty components [30]. High total protein intake in the usual diet might guard against frailty regardless of the source of protein and the amino acid composing the protein [31]. In a large cohort study of 24,417 healthy women aged 65–79 years, women with high protein intake were significantly less likely to develop frailty during follow-up. In fact, a 20% increase in protein intake (independent of the protein source) was related to a 32% reduction in frailty incidence [32]. Although we observed insufficient protein intake in the frail lung cancer patients, we failed to reveal the independent effect of protein deficiency on frailty in logistic regression analysis. Energy also plays a critical role in frailty development. 4731 American participants older than 60 years from the Third National Health and Nutrition Examination Survey (NHANES III) illustrated that the daily energy intake was the lowest in people who were frail, followed by pre-frail, and it was the highest in people who were not frail, independently of BMI [33]. Data from 802 persons aged 65 years and older participating in the InCHINANTI study also showed that frailty was associated with a daily energy intake of less than 21 kcal/kg [9]. However, the lung cancer patients ingested 18.89 kcal/kg energy in our study, which was lower than the recommended daily intake. Micronutrients intake may also have a direct association with frailty. Low intake or low plasma levels of carotenoids, selenium, magnesium, folate, vitamin C, vitamin E, *n*-3 fatty acids, and total polyphenols were found to be related to frailty syndrome or frailty criteria [34]. Additionally, the number of micronutrient deficiencies is also important and has been consistently associated with frailty. In a cross-sectional analysis of 802 persons in the InCHINANTI study, a low intake of more than three nutrients was associated with prevalent frailty, even independently of energy intake [9]. In our research, twenty nutrients were found to be remarkably lower in the frail patients, suggesting the higher risk of frailty, but only carbohydrate, calcium, and selenium intake deficiency were associated with frailty. An intervention trial showed that supplementation with essential amino acids and carbohydrates over 28 days of bedrest improved muscle mass loss [35]. The role of calcium on frailty have been attributed to the alteration in calcium and vitamin D metabolism on muscle health. Nutritional supplements containing 20 g protein, 24.2 g carbohydrate, 13 g lipids, 3 g dietary fiber, 500 IU vitamin D, and 480 mg calcium and physical activity co-interventions improved the functional status of institutionalized, frail, older adults [36]. Sarcopenic subjects showed a lower intake of selenium compared to the non-sarcopenic controls [37], which was in accordance with our results, but the contradictory results from logistic regression analysis that it was a risk factor for frailty still need to be clarified. Based on these findings, we speculated that the daily insufficiency of the various nutrients in lung cancer patients might be associated with frailty. Therefore, healthcare professionals should put emphasis on nutrition education for the patients to increase their daily dietary intake to decrease the risk of frailty.

Since diets are consumed in complex combinations of foods and nutrients, evaluating the association of dietary patterns with the risk of frailty in lung cancer patients may present a comprehensive picture of dietary effects on the risk of frailty, for which the Healthy Eating Index (HEI), the Alternative Healthy Eating Index (aHEI), World Cancer Research Fund/American Institute for Cancer Research (WCRF/AICR) guidelines, CHEI, and other indexes have been designed as the tools. A cross-sectional and longitudinal analysis in the population-based Rotterdam Study demonstrated that adherence to the national dietary guidelines was associated with lower frailty at baseline, and higher adherence was associated with lower frailty scores over time [38]. In a prospective cohort study, the Western diet was directly related to higher risk of two components of frailty: slow walking speed and weight loss [39]. On the other hand, adherence to the Mediterranean diet was associated with a lower risk of frailty [40]. Results from a variety of researches also indicated a better dietary quality was associated with lower risk of frailty. In the Nurses’ Health Study, higher adherence to the aHEI2010 was associated with a significantly reduced risk of frailty [10]. Similarly, in a cross-sectional analysis conducted in Japan, a dose–response relationship was observed between adherence to the Spinning Top score based on the Japanese Food Guidelines and the prevalence of frailty, with higher adherence being linked to a low prevalence of frailty [41]. In our study, we only found that frail lung cancer patients had lower CHEI total score, dairy score, sodium score, and cooking oils score than non-frail patients, without significant differences related to other components, and the total CHEI score was associated with frailty status. The above results suggested that improving overall dietary quality rather than focusing on individual nutrients or food groups might be beneficial for the frail patients.

Leucine, the primary precursor for activating muscle protein synthesis, plays a crucial role in regulating frailty status by activating mammalian target of rapamycin complex (mTORC) pathway [42]. Researches indicated that alterations in plasma amino acid profiles, including leucine, are associated with poor muscle mass [43]. In the present study, we observed that the frail patients had lower leucine intake compared to the non-frail patients, with higher plasma leucine levels. This might be attributed to the frail patients being in a state of metabolic synthesis resistance, leading to the release of more leucine to overcome the resistance and stimulate muscle protein synthesis [44].

The gut microbiota, a potential target to modulate health, could be shaped by different factors, and diet is one of the major influences. Short-term dietary modifications have been shown to have significant effects on microbial metabolites and host physiology [45]. Thus far, research on the associations among diet, gut microbiota, and frailty is limited. Adherence to the Mediterranean diet pattern has been associated with an increase in certain beneficial bacteria, which was positively correlated with a lower risk of frailty [46]. A randomized clinical trial also revealed that interventions with prebiotics demonstrated a significant improvement in frailty indicators such as exhaustion and muscle strength [47]. Deprivation of the gut microbiota resulted in decreased muscle mass in germ-free mice due to higher protein degradation than protein synthesis [18]. In our study, we failed to observe a significant difference in microbial alpha diversity between the frail and non-frail patients, which was in accordance with the previous study that also showed no statistical difference on the Shannon and Simpson indexes between the frailty and the control groups [48]. However, we demonstrated lower gut microbiota beta diversity in the frail patients. In individuals with sarcopenia, characterized by accelerated loss of muscle mass and function, a significant reduction in microbial beta diversity was confirmed [49]. Actinobacteriota, an important microbe in regulating inflammation [50] and subsequent reduction in skeletal muscle mass and function [51], was found to be increased in the frail lung cancer patients. Gordonibacter [52], characterized by its ability to produce urolith protein through polyphenol metabolism, was increased in the frail patients, while the relative abundance of Bacteroidota was decreased. Some species of Bacteroidota may play beneficial roles in the gut by metabolizing polysaccharides and oligosaccharides to provide nutrition and vitamins to the host and other intestinal microbial residents as well as pathogenic roles in other body locations [53]. Bacteroides fragilis gnotobiotic mice showed higher functional and muscle mass compared to germ-free mice [54]. However, the underlying mechanism of the interaction between diet and microbes and the role of these microbes in frailty development is not clear and still needs to be clarified.

This study represents the first investigation into the relationships between frailty status, diet, and gut microbiota in lung cancer patients. Although the study has several strengths, there are some limitations that should be acknowledged. First, the study is a cross-sectional design, and the causal relationship between frailty and nutrition cannot be evaluated directly because of the inability to evaluate temporal relationships in the data. Reverse causation could be possible, as dietary consumption and nutritional status might have been altered when participants started to be frail. Second, dietary data were recorded using 3-day, 24-h dietary recalls, so day-to-day variation could not be measured, and food intake could have changed during the research, and therefore, recall data might not represent participants’ long-term dietary patterns. Third, considering the influence of chemotherapy on the gut microbiota and appetite, blood and fecal samples were collected prior to chemotherapy. Accordingly, the available number of the samples was only 83 for blood and 50 for feces. Further longitudinal studies are necessary to uncover the trajectory of frailty in lung cancer patients and elucidate the role of diet in the development of frailty.

## 5. Conclusions

This study showed that the prevalence of frailty status in lung cancer patients was 32.5%. Compared with the non-frail patients, the frail patients had lower nutrients intakes and a poorer dietary quality, accompanied by gut microbiota dysbiosis. Among all the nutrients, carbohydrate, calcium, and selenium intake were associated with frailty. A healthy diet indicated by a higher total CHEI score might protect lung cancer patients from frailty. In addition, the frail patients had a lower dairy score, sodium score, and cooking oils score than the non-frail patients. Therefore, healthcare professionals should focus on nutrition education, such as encouraging patients to consume more dairy products and a proper amount of cooking oil and sodium.

## Figures and Tables

**Table 1:** Criteria for the definition of frailty developed by Fried et al.

The Fried Frailty Criteria	Detailed Description			
Non-expected weight loss	Unintentional loss of 4.5 kg (10 lb) in the year before the current evaluation or unintentional weight loss of at least 5% of the previous year’s body weight.			
Self-reporting fatigue	Evaluation of two statements of the CES-D scale: (a) I felt that everything I did was an effort; (b) I could not get going. Criterion positive if at least one condition is present for 3 days or more during the last week.			
Muscle weakness	Grip strength of the dominant hand, using hand-held dynamometer:			
	BMI/male	Cutoff (kg)	BMI/female	Cutoff (kg)
	≤24	≤29	≤23	≤17
	24–26	≤30	23–26	≤17.3
	26–28	≤30	26–29	≤18
	>28	≤32	>29	≤21
Low gait	Cutoff for time to walk 4.57 m at usual pace (static protocol):			
	Height/male (cm)	Cutoff (s)	Height/female (cm)	Cutoff (s)
	≤173	≥7	≤159	≥7
	>173	≥6	>159	≥6
Low physical activity level	Assessed by weekly energy expenditure by using the IPAQ-SF: 1 week’s activity < 600 MET-min/week.			
CES-D, Center for Epidemiological Studies Depression; IPAQ-SF, International Physical Activity Questionnaire—Short Form.				

**Table 2:** Characteristics of the frail and non-frail lung cancer patients.

	Frailty (*n* = 75)	Non-Frailty (*n* = 156)	*p*-Value
Age (years) ^1^	67.0 (61.0, 71.0)	64.0 (59.0, 69.0)	0.025
BMI (kg/m^2^) ^1^	20.9 (19.5, 23.6)	23.6 (21.8, 29.5)	<0.001
Sex, *n* (%) ^2^			
Male	59 (78.7)	116 (74.4)	0.474
Female	16 (21.3)	40 (25.6)	
Marital status, *n* (%) ^2^			
Married	69 (92.0)	142 (91.0)	0.931
Widowed/divorced/single	6 (8.0)	14 (9.0)	
Education level, *n* (%) ^2^			
Primary school or lower	25 (33.3)	46 (29.5)	0.812
Middle school	37 (49.3)	80 (51.3)	
High school/secondary school	8 (10.7)	22 (14.1)	
Junior college or higher	5 (6.7)	8 (5.1)	
Residence, *n* (%) ^2^			
Rural areas	22 (29.3)	31 (19.9)	0.274
Towns	12 (16.0)	27 (17.3)	
Urban areas	41 (54.7)	98 (62.8)	
Family monthly income (RMB), *n* (%) ^2^			
<2000	16 (21.3)	16 (10.3)	0.074
2000–5000	53 (70.7)	125 (80.1)	
>5000	6 (8.0)	15 (9.6)	
Smoking status, *n* (%) ^2^			
Never	10 (13.3)	23 (14.7)	0.774
Former/current	65 (86.7)	133 (85.3)	
Drinking status, *n* (%) ^2^			
Never	11 (14.7)	25 (16.0)	0.790
Former/current	64 (85.3)	131 (84.0)	
Presence of comorbidities, *n* (%) ^3^			
Yes	38 (50.7)	65 (41.7)	0.644
No	37 (49.3)	91 (58.3)	
Surgery, *n* (%) ^3^			
Yes	43 (57.3)	101 (64.7)	0.276
No	32 (42.7)	55 (35.3)	
Pathology type, *n* (%) ^3^			
Adenocarcinoma	60 (80.0)	117 (75.0)	0.644
Squamous cell carcinoma	12 (16.0)	33 (21.2)	
Small cell lung cancer	3 (4.0)	6 (3.8)	
Cancer stage, *n* (%) ^2^			
I	3 (4.0)	17 (10.9)	0.023
II	6 (8.0)	22 (14.1)	
III	12 (16.0)	37 (23.7)	
Ⅳ	54 (72.0)	80 (51.3)	

Data are shown as *n* (%) or median (25th, 75th percentile). ^1^ Mann–Whitney U test; ^2^ chi-square test; ^3^ Fisher’s exact test. BMI, body mass index.

**Table 3:** Nutrient intakes of the frail and non-frail lung cancer patients.

	Frailty (*n* = 75)	Non-Frailty (*n* = 156)	*p*-Value
Energy (kcal/d) ^1^	1080.0 (907.0, 1284.0)	1196.0 (1042.5, 1493.0)	<0.001
Protein (g/d) ^1^	51.6 (41.1, 61.2)	62.1 (46.9, 71.1)	<0.001
Fat (g/d) ^1^	37.4 (28.8, 44.5)	38.5 (31.1, 51.1)	0.141
Dietary fiber (g/d) ^1^	7.6 (5.1, 9.1)	8.5 (6.2, 12.4)	0.003
Carbohydrate (g/d) ^1^	128.2 (105.7, 155.8)	151.4 (128.1, 189.2)	<0.001
Cholesterol (mg/d) ^1^	482.0 (256.0, 672.0)	494.0 (327.3, 699.3)	0.331
Vitamin A (µgRAE/d) ^1^	346.0 (218.0, 517.0)	383.0 (291.5, 576.7)	0.093
Vitamin D (µg/d) ^1^	0.9 (0.0, 1.8)	1.3 (0.5, 4.5)	0.031
Vitamin E (mg/d) ^1^	10.7 (8.1, 15.2)	12.4 (9.1, 17.5)	0.083
Vitamin B1 (mg/d) ^1^	0.5 (0.4, 0.7)	0.6 (0.4, 0.8)	0.009
Vitamin B2 (mg/d) ^1^	0.7 (0.6, 1.1)	0.8 (0.7, 1.2)	0.026
Vitamin B6 (mg/d) ^1^	0.1 (0.0, 0.2)	0.1 (0.0, 0.2)	0.027
Vitamin C (mg/d) ^1^	74.6 (39.7, 124.5)	100.2 (60.2, 154.3)	0.003
Folate (µg/d) ^1^	89.7 (52.4, 129.0)	100.4 (66.7, 150.3)	0.095
Niacin (mg/d) ^1^	10.4 (7.8, 13.1)	12.3 (9.5, 15.5)	0.001
Biotin (µg/d) ^1^	2.6 (1.0, 4,9)	2.5 (1.2, 5.6)	0.525
Calcium (mg/d) ^1^	418.0 (266.0, 563.0)	527.0 (371.3, 686.0)	<0.001
Phosphorus (mg/d) ^1^	715.3 (571.8, 821.4)	830.3 (681.5, 997.2)	<0.001
Potassium (mg/d) ^1^	1308.8 (967.6, 1542.3)	1552.4 (1179.7, 1904.9)	<0.001
Sodium (mg/d) ^2^	4158.9 ± 1476.5	4211.6 ± 1490.4	0.788
Magnesium (mg/d) ^1^	179.0 (156.0, 214.3)	234.0 (178.5, 295.8)	0.001
Iron (mg/d) ^1^	12.7 (10.4, 16.4)	14.6 (11.3, 18.5)	0.017
Iodine (µg/d) ^1^	21.5 (14.1, 33.9)	26.2 (18.1, 36.8)	0.027
Zinc (mg/d) ^1^	6.7 (5.6, 8.4)	7.9 (6.3, 9.7)	0.001
Selenium (µg/d) ^1^	35.4 (23.4, 54.3)	41.6 (30.7, 61.5)	0.019
Copper (mg/d) ^1^	0.9 (0.7, 1.3)	1.1 (0.8, 1.6)	0.018
Manganese (mg/d) ^1^	2.6 (2.1, 3.5)	3.2 (2.5, 4.3)	0.002
Leucine (mg/d) ^1^	3494.9 (2955.2, 4178.6)	4190.0 (3308.8, 5439.4)	<0.001

Data are shown as median (25th, 75th percentile) or mean ± SD. ^1^ Mann–Whitney U test; ^2^ independent samples *t*-test.

**Table 4:** Multivariate logistic regression analysis of nutrients factors influencing frailty in the lung cancer patients (*n* = 231).

Variables	OR	95% CI	*p-*Value
Energy	1.00	0.99, 1.00	0.149
Protein	0.98	0.93, 1.03	0.443
Dietary fiber	1.03	0.91, 1.17	0.654
Carbohydrate	0.97	0.96, 0.99	0.003
Niacin	0.93	0.83, 1.05	0.223
Calcium	0.99	0.99, 1.00	0.025
Phosphorus	1.00	0.99, 1.00	0.439
Potassium	1.00	0.99, 1.00	0.346
Magnesium	1.00	0.99, 1.01	0.636
Iodine	0.99	0.98, 1.01	0.477
Zinc	0.88	0.65, 1.18	0.399
Selenium	1.03	1.00, 1.06	0.022
Copper	0.70	0.40, 1.21	0.201
Manganese	0.83	0.63, 1.09	0.172

Multiple logistic regression was used after adjusting for age, sex, cancer stage, and BMI.

**Table 5:** CHEI component scores of the frail and non-frail lung cancer patients.

	Frailty (*n* = 75)	Non-Frailty (*n* = 156)	*p-*Value
Total CHEI score ^1^	55.9 ± 8.3	60.4 ± 8.2	<0.001
Total grains ^2^	4.4 (3.2, 5.0)	4.7 (3.4, 5.0)	0.200
Whole grains and mixed beans ^2^	0.0 (0.0, 1.7)	0.0 (0.0, 1.6)	0.942
Tubers ^2^	0.0 (0.0, 3.0)	0.0 (0.0, 1.6)	0.504
Total vegetables ^2^	3.8 (2.1, 5.0)	3.8 (2.5, 5.0)	0.410
Dark vegetables ^2^	2.9 (0.5, 5.0)	3.1 (1.7, 5.0)	0.168
Fruits ^2^	10.0 (6.4, 10.0)	10.0 (10.0, 10.0)	0.137
Poultry ^2^	0.0 (0.0, 0.0)	0.0 (0.0, 0.0)	0.128
Red meat ^2^	3.4 (2.2, 5.0)	3.6 (2.2, 4.9)	0.952
Fish and seafood ^2^	5.0 (0.0, 5.0)	5.0 (0.0, 5.0)	0.225
Eggs ^2^	5.0 (0.0, 5.0)	5.0 (4.6, 5.0)	0.609
Dairy ^2^	0.0 (0.0, 5.0)	5.0 (0.0, 5.0)	0.005
Soybeans ^2^	0.0 (0.0, 4.0)	0.0 (0.0, 5.0)	0.833
Seed and nuts ^2^	0.0 (0.0, 0.0)	0.0 (0.0, 0.0)	0.636
Cooking oils ^2^	10.0 (10.0, 10.0)	10.0 (10.0, 10.0)	0.022
Sodium ^2^	0.0 (0.0, 2.5)	1.2 (0.0, 3.8)	0.021
Alcohol ^2^	5.0 (5.0, 5.0)	5.0 (5.0, 5.0)	1.000
Added sugars ^2^	5.0 (5.0, 5.0)	5.0 (5.0, 5.0)	0.326

Data are shown as median (25th, 75th percentile) or mean ± SD. ^1^ Independent sample *t*-test; ^2^ Mann–Whitney test. CHEI, Chinese Healthy Eating Index.

**Table 6:** Logistic regression analysis of CHEI and its components influencing frailty in the lung cancer patients (*n* = 231).

Variables	OR	95% CI	*p-*Value
Univariate			
Total CHEI score	0.94	0.91, 0.97	<0.001
Dairy	0.84	0.75, 0.94	0.002
Sodium	0.89	0.79, 1.00	0.055
Cooking oils	0.07	0.01, 1.25	0.070
Multivariate			
Total CHEI score	0.94	0.90, 0.99	0.009
Dairy	0.91	0.79, 1.05	0.214

Multiple logistic regression was used after adjusting for age, sex, cancer stage, and BMI. CHEI, Chinese Healthy Eating Index.

## Data Availability

The data presented in this study are available from the corresponding author upon request.

## Supplementary Materials

The following supporting information can be downloaded at: https://www.mdpi.com/article/10.3390/nu15194298/s1, Table S1: Univariate logistic regression analysis of nutrients factors influencing frailty in the lung cancer patients (*n* = 231).
