# Supplementary material for: Diet Is Associated with Frailty in Lung Cancer: A Possible Role of Gut Microbiota
Source: Nutrients. 2023 Oct 9;15(19):4298. doi: 10.3390/nu15194298 (PMC10574134; doi:10.3390/nu15194298)
Supplement: Supplementary file 1 [file nutrients-15-04298-s001.zip › nutrients-2623592-supplementary.pdf]

Table S1. Univariate logistic regression analysis of nutrients factors influencing frailty in the lung cancer patients (n = 231).

**Variables**	**OR**	**95% CI**	***p*-Value**
Energy	0.99	0.98, 0.99	< 0.001
Protein	0.97	0.96, 0.99	< 0.001
Carbohydrate	0.99	0.98, 0.99	< 0.001
Dietary fiber	0.90	0.83, 0.97	0.004
Vitamin A	1.00	0.99, 1.00	0.986
Vitamin B1	1.01	0.97, 1.04	0.682
Vitamin B2	0.90	0.67, 1.21	0.503
Vitamin B6	1.00	0.99, 1.01	0.906
Vitamin C	0.99	0.99, 1.00	0.064
Niacin	0.90	0.84, 0.96	0.002
Calcium	0.99	0.99, 0.99	< 0.001
Phosphorus	0.99	0.99, 0.99	< 0.001
Potassium	0.99	0.99, 0.99	< 0.001
Magnesium	0.99	0.98, 0.99	< 0.001
Iron	0.97	0.92, 1.02	0.194
Zinc	0.81	0.72, 0.92	< 0.001
Iodine	0.97	0.95, 0.99	0.017
Selenium	0.99	0.98, 0.99	0.026
Copper	0.71	0.51, 0.98	0.035
Manganese	0.78	0.64, 0.95	0.013
